# Maternal Dietary Carbohydrate Intake and Newborn Aortic Wall Thickness

**DOI:** 10.3390/nu13041382

**Published:** 2021-04-20

**Authors:** Kirsty M. Mckenzie, Reeja Nasir, Yang Kong, Hasthi U. Dissanayake, Rowena McMullan, Adrienne Gordon, Alice Meroni, Melinda Phang, Michael R. Skilton

**Affiliations:** 1 Boden Collaboration for Obesity, Nutrition, Exercise & Eating Disorders, Charles Perkins Centre, University of Sydney, Camperdown, NSW 2006, Australia; k.mckenzie@sydney.edu.au (K.M.M.); reeja.nasir@sydney.edu.au (R.N.); yangkong0310@hotmail.com (Y.K.); hasthi.dissanayake@sydney.edu.au (H.U.D.); rowenamcm@gmail.com (R.M.); alicemeroni90@gmail.com (A.M.); melinda.phang84@gmail.com (M.P.); 2 Sydney Medical School, Charles Perkins Centre, University of Sydney, Camperdown, NSW 2006, Australia; adrienne.gordon@sydney.edu.au; 3 Royal Prince Alfred Hospital, Missenden Road, Camperdown, NSW 2050, Australia; 4 Sydney Institute for Women, Children and their Families, Sydney Local Health District, Sydney, NSW 2000, Australia

**Keywords:** cardiovascular disease, aortic intima-media thickness, maternal diet

## Abstract

Evidence from animal models indicates that maternal diet during pregnancy affects offspring cardiometabolic health. Improving carbohydrate quality during high-risk pregnancies reduces aortic intima-medial thickness; a marker for early atherosclerosis; in the infant offspring. We sought to determine whether maternal carbohydrate quantity and quality are associated with newborn aortic intima-medial thickness in healthy pregnancies. Maternal diet throughout pregnancy was evaluated in 139 mother–child dyads using a validated food frequency questionnaire. Carbohydrate intake was expressed as quantity (% total energy), quality (fibre, glycaemic index), and glycaemic burden (glycaemic load). Aortic intima-medial thickness was measured by high-frequency ultrasound of the neonatal abdominal aorta. Neither quantity nor quality of maternal carbohydrate intake during pregnancy was associated with meaningful differences in offspring maximum aortic intima-medial thickness with the exception of fibre intake in women with overweight or obesity which was inversely associated (−8 μm [95% CI −14, −1] per g fibre, *p* = 0.04). In healthy pregnancy, the quantity and quality of maternal carbohydrate intake is likely not a meaningful modifiable lifestyle factor for influencing offspring vascular health. The effect of carbohydrate quality may only be evident in high-risk pregnancies, consistent with previous findings. These findings may be confirmed in prospective dietary trials in pregnancy.

## 1. Introduction

Cardiovascular disease (CVD) is one of the leading causes of morbidity and mortality globally. Atherosclerosis is the underlying cause for the majority of heart attacks and ischemic strokes [1]. Despite most clinical CVD events occurring in the 5th decade of life and later, the pathogenesis of atherosclerosis is initiated in utero [2]. Accordingly, a life course approach to reduce CVD risk has marked potential yet remains poorly understood. Pre-clinical markers of atherosclerosis, such as arterial intima-media thickness (IMT), are an established means by which to study cardiovascular risk and interventions [3], and may be particularly relevant for identifying early-life risk factors.

Nutrition-related characteristics, including impaired foetal growth and maternal obesity, are key early-life risk factors for later life cardiovascular disease and increased arterial IMT in childhood [4,5]. Maternal dietary risk factors remain poorly characterised. Carbohydrate intake comprises a significant component of most Westernised diets [6]. Given that glucose is the primary energy substrate towards fetal growth, maternal dietary carbohydrate intake and glycaemic burden are important fetal exposures [7]. Both dietary glycaemic index (GI), a measure of carbohydrate quality, and glycaemic load (GL), a measure of overall glycaemic burden, are strong determinants of glucose levels throughout pregnancy [8]. We have previously shown that the infant offspring of women with a high-risk pregnancy who were randomly allocated to consume a low GI during pregnancy had lower aortic IMT [9], and that higher maternal glycaemic index and lower fibre intake in women with healthy pregnancies are associated with poorer measures of cardiovascular control in their newborn offspring [10]. It is not known whether maternal carbohydrate quantity or quality are associated with aortic IMT infants from healthy pregnancies.

Accordingly, we sought to determine whether the quantity of maternal carbohydrate intake, measured as percentage total energy intake, the quality of maternal carbohydrate, measured as GI and fibre intake, and overall glycaemic burden, measured as GL, in healthy pregnancies are associated with aortic IMT in their newborn offspring.

## 2. Materials and Methods

### 2.1. Participant Characteristics

The cohort in this manuscript was part of a larger study exploring the associations of infant body fatness with offspring cardiovascular risk [5]. Mothers and their newborns were recruited from the postnatal wards of Royal Prince Alfred Hospital (Sydney, Australia). Singleton newborns with gestational age greater than 34 weeks and who had undergone a body composition measurement shortly after birth were eligible for the study. Newborns from multiple birth pregnancy, those with significant congenital abnormalities and those requiring ongoing intensive care were excluded from the study. This study was conducted in accordance with ethical standards and ethical approval was granted from the Sydney Local Health District Human Research Ethics Committee (HREC/14/RPAH/478). Participation was voluntary and informed written consent was obtained from all mothers.

Of the 224 newborns recruited, maternal dietary data was available for 214 and of those aortic IMT was available from 179 infants. Mothers with diabetes (*n* = 3), gestational diabetes mellitus (GDM) (*n* = 35), preeclampsia (*n* = 8) and hypertension of pregnancy (*n* = 6) were excluded from this analysis, leaving 139 participants.

Maternal demographic and perinatal characteristics were collected using a self-administered questionnaire and confirmed using health records. An electronic food frequency questionnaire, the Cancer Council Victoria Dietary Questionnaire for Epidemiological Studies Version 2 (DQESV2), was used to capture maternal dietary intake during pregnancy. The DQESV2 covers 74 food and beverage items typically consumed in Australia, grouped according to several categories including cereal foods, sweets and snacks, dairy products, meats and fish, and fruit and vegetables. Nutrient intakes are derived using the Australian Food Composition Database (NUTTAB95) [11,12]. When completing the dietary questionnaire, women were requested to consider their dietary intake throughout their pregnancy, which we have validated using dietary biomarkers [13].

Physical activity during pregnancy was assessed using a self-administered validated questionnaire which instructs respondents to report time spent doing a particular activity [14]. Total activity was calculated as metabolic equivalent (MET) x hours per week as per the protocol described in Chasan-Taber et al. (2004) [14].

Other birth and pregnancy data were collected as part of routine clinical care, these were then obtained by the study team from health records. Aortic IMT was assessed as per best practice guidelines [15]. The far-wall of the neonatal abdominal aorta was imaged using high-frequency B-mode ultrasonography (EPIQ 5, Phillips Medical Systems, Bothell, WA, USA) using a linear array probe (18–5 MHz). Aortic IMT was subsequently measured off-line using a validated semi-automated edge-detection software, Carotid Analyzer for Research (Version 5, Medical Imaging Applications, Coralville, IA, USA), by a blinded assessor (Y.K.). Maximum aortic IMT was used for all analyses as it has been shown to have the strongest associations with risk factors in early life [15]. The final IMT value was the mean maximum thickness from a minimum three end-diastolic frames as previously described [5].

### 2.2. Statistical Analysis

Descriptive data are presented as mean (SD) for continuous variables and *n* (%) for categorical variables, unless otherwise stated. Visual assessment and Kolmogorov–Smirnov tests were used to assess data for normality and non-parametric data were log-transformed.

Absolute maternal carbohydrate intake during pregnancy (g/d) was converted to energy content (kJ/d) using a conversion factor of 17 kJ per gram of carbohydrate [16], and subsequently converted to a percentage of daily energy intake (%) for statistical analysis. Total fat (and fatty acids) and protein were similarly converted to percentage daily energy intake with a conversation factor of 37 kJ and 17 kJ per gram, respectively [16]. Maternal carbohydrate intake, GI, GL and fibre were analysed both as continuous variables and as categorical variables based on quartiles. The range and cut-offs for quartiles were as follows: carbohydrate intake (minimum 30.3% total energy intake; 25th percentile 40.5%; 50th percentile 42.9%; 75th percentile 47.4%; maximum 62.8%); GI: (minimum 41.7; 25th percentile 47.0; 50th percentile 49.8; 75th percentile 52.0; maximum 59.8); fibre: minimum 3.5 g/d; 25th percentile 17.3 g/d; 50th percentile 20.8 g/d; 75th percentile 26.8 g/d; maximum 65.3 g/d. Quartiles for GL were calculated using the residual method, adjusted for maternal total energy intake [17].

Statistical analysis was performed with SPSS Statistics (Version 26; IBM Corp., Somers, NY, USA). Results were considered significant at 2p < 0.05. Unadjusted correlations were undertaken using Pearson’s and Spearman’s correlation for parametric and non-parametric data, respectively. Multivariable linear regression was performed to evaluate associations between maternal dietary characteristics and infant aortic IMT. Analyses were adjusted for maternal total energy intake during pregnancy, maternal age and newborn sex. An a priori power calculation had been carried out as part of the larger study based on infant body fatness [5]. For this cohort, the sample size (*n* = 139 mother–child dyads) provided 85% power to detect a correlation coefficient of 0.25 at 2p < 0.05.

## 3. Results

### 3.1. Demographics

Maternal and neonatal characteristics are summarised in Table 1. Mothers who participated in the study had a mean age of 33.6 years [SD 4.4]. On average, women obtained 43.5% (SD 5.4) of their total energy intake from carbohydrates. While GI was relatively low, fibre intake was below the current recommended intake for pregnant women in Australia [18]. The mean macronutrient proportions (Carbohydrate:Fat:Protein) when stratified by quartiles of carbohydrate intake were: Q1 37:42:22; Q2 41:39:20; Q 3 45:37:19 and Q4 50:33:18.

### 3.2. Infant Aortic Intima-Medial Thickness and Maternal Carbohydrate Intake

**Figure 1:**
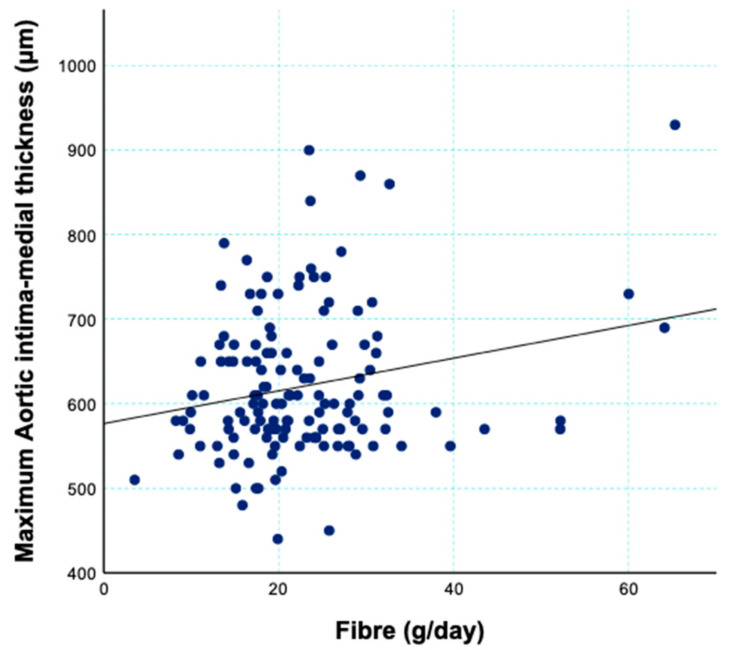
Correlation between maternal fibre (g/d) intake and offspring maximum aortic intima-medial thickness.

On univariate analysis, maternal fibre intake (r = 0.219, *p* = 0.010; Figure 1) was positively associated with offspring aortic IMT whilst carbohydrate intake (r = 0.089, *p* = 0.30), glycaemic index (r = 0.040, *p* = 0.64) and glycaemic load (r = 0.131, *p* = 0.12) were not. In multivariable models adjusted for total energy intake, maternal age and newborn sex, neither the quality nor quantity of maternal carbohydrate intake was associated with meaningful differences in offspring aortic IMT. These findings were similar when the carbohydrate characteristics were expressed as continuous outcomes (9 μm (−4, 22) per 5% energy from carbohydrate, *p* = 0.19; 1 μm (−20, 22) per 5 units GI, *p* = 0.91; 48 μm (−18, 114) per unit log-GL, *p* = 0.18; 2 μm (−1, 5) per g fibre, *p* = 0.17), or in quartiles of intake (Table 2). Further adjustment for maternal BMI, maternal physical activity during third trimester, and infant aortic diameter did not modify these associations (results not shown).

In analyses stratified by maternal BMI, there was a positive association of dietary fibre intake with offspring aortic IMT in mothers with heathy BMI (<25 kg/m^2^) although this did not reach statistical significance (3 μm (−0, 6) per g fibre, *p* = 0.10); whereas there was evidence for an inverse association of fibre with aortic IMT in women with overweight or obesity (*n* = 27; −8 μm (−14, −1) per g fibre, *p* = 0.04).

In post hoc analysis of carbohydrate intake expressed as grams per day, there was a strong association with aortic IMT (0.634 (0.166, 1.101), *p* = 0.008; adjusted for total energy intake, maternal age and newborn sex). This association remained significant after further adjustment for maternal intake of sugars (0.638 (0.063, 1.214), *p* = 0.030).

In additional post hoc analysis, total fat, fatty acids classes (saturated, monounsaturated and polyunsaturated acids) and protein % daily energy intake were explored as dietary exposures. Neither total fat (r = −0.123, *p* = 0.151) nor protein (r = 0.018, *p* = 0.833) were significantly correlated with infant aortic IMT in crude correlation analysis, nor in multivariable regression (3 μm (−6, 1) per % energy from total fat, *p* = 0.11; 0 μm (−5, 5) per % energy from protein, *p* = 0.94; adjusted for total energy intake, maternal age and newborn sex). Associations of fatty acid classes with aortic IMT were not significant (results not shown).

## 4. Discussion

Our findings indicate that predominantly neither the quantity nor quality of maternal carbohydrate intake are associated with meaningful differences in aortic IMT in the offspring of women with a metabolically healthy pregnancy. However, there was some evidence that dietary fibre intake was associated with lower offspring aortic IMT in women with overweight or obesity.

Carbohydrates are the major source of energy in most diets [19]. Both the quantity of carbohydrates in the diet and their quality are associated with maternal blood glucose levels and pregnancy outcomes [20]. It has been previously demonstrated that the infants of women with a high-risk pregnancy and who were randomly assigned to a low glycaemic index diet, consistent with higher quality carbohydrates, showed no difference in newborn body fatness or birth weight, compared to controls. However, at 1 year of age, these infants of women assigned to the low glycaemic index diet had reduced aortic IMT [21], suggesting that carbohydrate quality may impact infant vascular development. Interestingly, the control group in this trial was assigned a high fibre diet. Our current finding of a direct association of fibre with aortic IMT in unadjusted correlation analysis is consistent with this previous finding, and may suggest a counterintuitive adverse effect of maternal fibre intake on the onset and early progression of atherosclerosis in the offspring.

We previously demonstrated that maternal carbohydrate intake during pregnancy was not significantly correlated with newborn body fatness or infant birth weight, although there is a weak association of carbohydrate quality, as measured by fibre and GI, with offspring cardiac autonomic function [10]. This highlighted a novel putative link between maternal diet and infant cardiovascular risk. In this study, we aimed to further explore this link by measuring offspring aortic IMT, an age-appropriate surrogate marker for atherosclerotic burden [15]. While we did not observe any meaningful associations with aortic IMT in multivariable models adjusted for appropriate covariates, it has been proposed that a longer time-course may be required for the development of aortic IMT in response to specific exposures [5]. This may at least partially explain the divergent results observed in the associations of cardiac autonomic activity and aortic IMT with carbohydrate quality, with the former being more rapidly affected by risk exposures.

In a post hoc analysis in which maternal carbohydrate intake was expressed in grams per day, adjusting for energy intake as a covariate, we did find a meaningful positive association with offspring aortic IMT. It may be that our a priori analysis of carbohydrate intake as a percentage of energy intake, with additional adjustment for energy as a covariate, over adjusts for energy intake.

It may be that any effects of carbohydrate quality on offspring vascular health are only evident in higher risk pregnancies, consistent with changes in dietary quality countering the vascular effects of poor metabolic health. Indeed, we have previously shown that the infant offspring of women with a high-risk pregnancy who were randomly allocated to consume a low GI diet during pregnancy had lower aortic IMT [9]. This is consistent with our subgroup analyses in women with overweight or obesity, in whom fibre is inversely associated with aortic IMT. Our main findings, that there are no meaningful association of maternal carbohydrate quality or quantity with offspring arterial wall thickness, may provide reassurance to women with healthy pregnancies, that their carbohydrate intake (within normal ranges) is unlikely to have a meaningful direct impact on their offspring’s cardiovascular health.

There are several strengths and limitations to this study. We used an FFQ validated in pregnant women [13], and to minimise the effect of mis-reporting of overall nutrient quantities we used measures that are proportionate to energy intake and analyses adjusted for total energy intake. The use of aortic IMT is the most age-appropriate method for assessing subclinical atherosclerosis during infancy and childhood [15], consistent with post-mortem studies showing that the abdominal aorta is the first site to develop atherosclerotic lesions [22]. As this is a cross sectional sample, we have not been able to assess potential longer-term programming of offspring cardiometabolic health, although this should be a priority for long term pregnancy and birth cohorts, which would also have greater statistical power than our current analysis. Carbohydrate characteristics were the focus of this manuscript and given the implications for modelling in an isocaloric setting and the small sample size, models were not adjusted for other macronutrients (i.e., fat and protein). Whilst our post hoc analysis of total fat, fatty acid classes and protein did not produce any meaningful associations with infant aortic IMT in crude correlations and multivariable regression, exploration of overall diet composition, including food-based analyses and complex nutrient interactions, are an area that requires future exploration. Psychosocial characteristics such as stress, anxiety and social support were not collected in this cohort, although they are known to affect health behaviors during pregnancy, including dietary intake [23]. In their study, Hurley et al. (2005) [23] showed that women who reported higher stress and anxiety levels during pregnancy increased their carbohydrate and fat intake, respectively. The association between psychosocial factors and diet in pregnancy is similar to what is otherwise observed in adults [24] and their influence should be considered in future research linking maternal diet with offspring cardiovascular outcomes. Gestational weight gain was not measured, and therefore we are unable to determine whether it is a potential mediator of these associations, or a confounder. We excluded women with gestational diabetes from our current analysis, due to the potential that their clinical dietary advice received during pregnancy may result in spurious associations. Our sample was recruited from a single site, with a diverse inner-city population albeit small and relatively affluent. Finally, our a priori sample size calculation was based on infant body fatness as the exposure. As such, our study may be potentially underpowered to detect weaker associations of maternal dietary exposures with offspring aortic IMT.

In conclusion, we find that quality and quantity of maternal carbohydrate intake are not meaningfully associated with newborn aortic IMT, with the exception of maternal fibre intake in women with overweight or obesity. Accordingly, the effects of maternal carbohydrate quality on offspring vascular health may only be evident in high-risk pregnancies. Future dietary trials and cohort studies applying validated and standardized methodologies could look to determine causality and longer-term associations, respectively.

## Figures and Tables

**Table 1:** Maternal, including diet, and neonatal characteristics.

Characteristic	
Maternal Demographics	
Age (years)	33.6 (4.4)
Height (cm)	164.9 (6.5)
Pre-pregnancy BMI (kg/m^2^)	22.8 (3.9)
Highest level of education completed (*n* (%))	
High School	15 (10.8)
More than High School	124 (89.2)
Ethnicity (*n* (%))	
Caucasian	89 (64.0)
Asian	30 (21.6)
South Asian	11 (7.9)
Middle Eastern	5 (3.6)
Other	4 (3.7)
Maternal smoking (*n* (%))	
Current	5 (3.6)
Never	128 (92.1)
Previous	6 (4.3)
Maternal Diet	
Total energy intake (kJ/d)	7786 (3828)
Carbohydrate intake (% total energy)	43.5 (5.4)
Carbohydrate (g/d)	197.5 (96.4)
Sugars (g/d)	89.7 (39.5)
Fat intake (% total energy)	37.7 (4.4)
Fat (g/d)	79.7 (41.3)
Protein intake (% total energy)	19.5 (2.8)
Protein (g/d)	90.0 (50.7)
Fibre (g/d)	22.7 (9.7)
Glycaemic Index	49.7 (5.1)
Glycaemic Load	99.0 (39.0)
Total energy expenditure (MET.hours/week)	284.5 (127.4)
Newborn	
Female/Male (*n* (%))	74 (55)/65 (45)
Gestational age (weeks)	38.7 (1.6)
Birth weight (g)	3339.6 (566.4)
Birth length (cm)	49.4 (2.6)
Head circumference (cm)	34.6 (1.5)
Maximum aortic IMT (μm)	618 (83)

Values are mean (SD) for continuous variables and *n* (%) for categorical variables. Glycaemic load was not normally distributed and is expressed as median (interquartile range). BMI, Body Mass Index; MET, metabolic equivalent; IMT, intima-media thickness.

**Table 2:** Associations between maternal carbohydrate intake, both quantity and quality, with newborn aortic intima-medial thickness (IMT).

	Aortic IMT (μm)	
	*n* = 139	
	β (95% CI)	*p* Value
Carbohydrate		
Q_1_	Reference	
Q_2_	−11 (−52, 30)	0.59
Q_3_	28 (−13, 69)	0.17
Q_4_	15 (−26, 55)	0.48
Glycaemic Index		
Q_1_	Reference	
Q_2_	11 (−30, 52)	0.61
Q_3_	−13 (−56, 29)	0.54
Q_4_	10 (−31, 51)	0.37
Glycaemic Load		
Q_1_	Reference	
Q_2_	−11 (−52, 30)	0.61
Q_3_	−12 (−52, 29)	0.58
Q_4_	18 (−23, 58)	0.39
Fibre		
Q_1_	Reference	
Q_2_	−6 (−46, 35)	0.79
Q_3_	21 (−22, 64)	0.34
Q_4_	7 (−44, 59)	0.78

Values are unstandardized β-regression coefficients (95% CI) from multivariable regression analyses and represent the differences in newborn maximum aortic MT (μm), adjusted for total energy intake, maternal age and newborn sex.

## Data Availability

The data presented in this study are available on request from the corresponding author. The data are not publicly available as participants of this did not consent for their data to be shared publicly.
